# Unexpected diversity found within benthic microbial mats at hydrothermal springs in Crater Lake, Oregon

**DOI:** 10.3389/fmicb.2022.876044

**Published:** 2022-09-14

**Authors:** Amanda Stromecki, Laura Murray, Heather Fullerton, Craig L. Moyer

**Affiliations:** ^1^Department of Biology, Western Washington University, Bellingham, WA, United States; ^2^Department of Biology, College of Charleston, Charleston, SC, United States

**Keywords:** hydrothermal springs, community structure, microbial mats, chemoautotrophs, Crater Lake

## Abstract

Crater Lake, Oregon is an oligotrophic freshwater caldera lake fed by thermally and chemically enriched hydrothermal springs. These vents distinguish Crater Lake from other freshwater systems and provide a unique ecosystem for study. This study examines the community structure of benthic microbial mats occurring with Crater Lake hydrothermal springs. Small subunit rRNA gene amplicon sequencing from eight bacterial mats was used to assess community structure. These revealed a relatively homogeneous, yet diverse bacterial community. High alpha diversity and low beta diversity indicate that these communities are likely fueled by homogeneous hydrothermal fluids. An examination of autotrophic taxa abundance indicates the potential importance of iron and sulfur inputs to the primary productivity of these mats. Chemoautotrophic potential within the mats was dominated by iron oxidation from *Gallionella* and *Mariprofundus* and by sulfur oxidation from *Sulfuricurvum* and *Thiobacillus* with an additional contribution of nitrite oxidation from *Nitrospira*. Metagenomic analysis showed that *cbbM* genes were identified as *Gallionella* and that *aclB* genes were identified as *Nitrospira*, further supporting these taxa as autotrophic drivers of the community. The detection of several taxa containing *arsC* and *nirK* genes suggests that arsenic detoxification and denitrification processes are likely co-occurring in addition to at least two modes of carbon fixation. These data link the importance of the detected autotrophic metabolisms driven by fluids derived from benthic hydrothermal springs to Crater Lake’s entire lentic ecosystem.

## Introduction

Crater Lake is an ultra-oligotrophic freshwater caldera lake in south-central Oregon at the crest of the Cascade Mountain range. It formed in the crater of Mount Mazama left behind after a volcanic eruption approximately 7,000 years ago, yet volcanic activity has taken place as recently as 4,000 years ago (Bacon et al., 2002). Crater Lake is known for its exceptionally clear water and deep basin; it is the ninth deepest lake in the world with a maximum depth of 594 meters (Collier et al., 1991; Bacon et al., 2002). Due to its clarity, Crater Lake receives significant ultraviolent light penetrance that limits the dissolved organic carbon and creates nutrient-poor conditions (Urbach et al., 2001). Although highly oxygenated, its primary productivity is also limited by nitrogen and trace metals (McManus et al., 1992; Groeger, 2007). The relatively small catchment area of the lake has no surface outlet and is fed primarily by rain and snowmelt, receiving minimal anthropogenic or allochthonous input of water (Page et al., 2004; Urbach et al., 2007). Due to these conditions, Crater Lake shares many similarities with oxygenated, oligotrophic ocean waters.

Crater Lake also resembles a marine system due to deep active hydrothermal springs fed by dense, enriched fluids that lie in the bottom of the lake. However, the thermally and chemically enriched hydrothermal springs distinguish Crater Lake from other freshwater systems and provide a unique ecosystem for study. These hydrothermal fluids are slightly elevated in temperature compared to the surrounding lake water, creating an inverse temperature gradient (McManus et al., 1996). The hydrothermal springs are also enriched in dissolved CO_2_ compared to bulk lake water (Collier et al., 1991). In addition, the springs contain elevated concentrations of dissolved iron, sulfur, and manganese ions, bringing critical nutrients to the oligotrophic environment (Collier et al., 1991). These vent-derived chemicals provide bioavailable nutrients that benthic microorganisms may utilize; therefore, hydrothermal input to Crater Lake is crucial for primary productivity (Urbach et al., 2001, 2007).

Previous research on the hydrothermal springs at the bottom of Crater Lake was conducted by researchers from Oregon State University using the research submersible Deep Rover (Drake et al., 1990). Surveys of the hydrothermal venting sites led to the discovery of benthic pools that formed in depressions in the lake floor and filled with chemically enriched hydrothermal fluids. Two primary pool locations were identified: Llao’s Bath in the south-central region of the lake, and Palisades Point Pools in the north-east basin (Collier et al., 1991). At both locations, dense microbial mats lined the pool margins and outlets (Dymond et al., 1989), suggesting the bacterial communities rely on these dense, nutrient-rich fluids emanating from the hydrothermal springs.

The benthic microbial mats that surround the pools and hydrothermal venting sites were hypothesized to be driven by chemoautotrophic bacteria (Dymond et al., 1989). Because chemoautotrophs obtain energy from inorganic chemicals such as those provided by hydrothermal vent fluids, they function as primary producers in environments lacking light or organic carbon (Nakagawa and Takai, 2008; McNichol et al., 2018). Crater Lake benthic microbial mats appeared to take advantage of the gradient among reduced forms of iron, sulfur, and manganese where hydrothermal fluids interact with the oxygenated freshwater lake (Dymond et al., 1989; Collier et al., 1991). These reduced ions provide an abundant energy source for chemoautotrophs that in turn produce organic carbon compounds utilized by other trophic levels, supporting diverse microbial communities (e.g., Sievert and Vetriani, 2012; Hager et al., 2017).

Cellular morphology was initially used to characterize these benthic microbial mat communities in Crater Lake, revealing a predominantly ubiquitous sheathed morphotype composition. Researchers used scanning electron microscopy to describe sheath-forming bacteria and identified the genera *Gallionella* and *Leptothrix* (Dymond et al., 1989). *Gallionella* and *Leptothrix* are found in freshwater environments and are capable of oxidizing iron for energy (Emerson et al., 2010). *Leptothrix* has also been shown to oxidize manganese ions (Eggerichs et al., 2020). Elevated levels of ferrous iron and manganese were detected at these mat sites (Dymond et al., 1989), supporting the hypothesis of mat communities dominated by iron-oxidizing chemoautotrophs (Bennett et al., 2014). Both are good candidates for functioning as primary producers within these microbial mats; however, many other genera also form sheaths and therefore microscopy is insufficient to accurately resolve mat community structure and diversity.

The Zetaproteobacteria are another more recently discovered class of iron-oxidizing bacteria capable of forming sheath-rich mat matrices (Emerson et al., 2007). Zetaproteobacteria habitat preferences have been best described in the submarine volcano Lō‘ihi Seamount where hydrothermal fluids rich in CO_2_ and iron and low in sulfide support dense mat communities (Glazer and Rouzel, 2009; Fullerton et al., 2017). Zetaproteobacteria have also been found in diffuse flow sites within high-temperature chimneys, the marine subsurface, brackish coastal environments, and CO_2_-rich terrestrial springs. Among these disparate environments, the shared habitat conditions for Zetaproteobacteria are brackish to hypersaline water, a supply of reduced iron, and micro-oxic conditions (McAllister et al., 2019). These conditions are present in the benthic hydrothermal springs found in Crater Lake, making Zetaproteobacteria an ideal candidate for an autotrophic, sheath-forming iron-oxidizer driving the primary production of these mat communities.

Since previous studies of the benthic microbial mats relied solely on morphology, further analysis is necessary to identify which genera of sheath-forming lithoautotrophs are present and to understand the community structure differences across the various mat locations. Amplicon sequencing targeting the small subunit (SSU) rRNA gene has been shown to be an efficacious tool to accurately identify community structure and diversity (Caporaso et al., 2012; Hugerth and Andersson, 2017). SSU rRNA gene amplicon sequencing can therefore be used to reveal the diversity within the microbial mats from hydrothermal springs and hypersaline pools in Crater Lake and to identify putative lithoautotrophs. These data can be interrogated to determine which operational taxonomic units (OTUs) are present in high abundances within the communities, revealing which OTUs are playing major ecological roles in these mats. Since the SSU rRNA gene is widely used, our resulting analyses can then be compared to previously described ecosystems dominated by other lithoautotrophs such as *Gallionella*, *Leptothrix,* and *Zetaproteobacteria* (Johnson et al., 2012; Hager et al., 2017). From this taxonomic information, we can infer the metabolic requirements of the microbial mats by identifying the autotrophs present in high abundances to determine if the communities are driven by iron oxidation. This can then be supported by shotgun metagenomics to verify that the autotrophic pathways are present in the taxa identified by SSU rRNA gene sequencing. These results will allow for insights into the potential for enhanced diversity of benthic microbial mats at hydrothermal springs in Crater Lake whose autotrophic members may affect crucial nutrients introduced to this oligotrophic ecosystem. These findings reveal patterns in mat community complexity and diversity at a stable, homogeneous hydrothermal system that can be compared to the episodic, heterogeneous conditions found at other hydrothermal vent ecosystems.

## Materials and methods

### Sample collection and location descriptions

Microbial mat and adjacent mat fluid collection occurred in the south-central and north-east regions of Crater Lake during 16 HOV Deep Rover dives in August 1989. Samples were collected from two primary areas: seven from Llao’s Bath/Brain Mat complex in the south-central and one from Palisades Point Pools in the north-east regions of Crater Lake. Sample names, sites, and map locations along with geochemical profiles of respective mat and pool fluids are described in Table 1. These samples were collected, and chemical analyses were done by Collier et al. (1991) during a detailed study of hydrothermal activity at the bottom of the lake. A 50 kHz echo sounder was used to determine the depths of mats and pools. Mat temperatures were collected using a temperature probe attached to the wrist mechanism of *Deep Rover’s* mechanical arm that was inserted into bacterial mat features. Water temperatures were collected using conductivity, temperature, and depth or “CTD” instrument carried on the submersible. Bacterial mat samples were collected using either push cores or Go-Flo bottles to collect mats and fluids with a minimum of lake water admixture (Dymond et al., 1989; Collier et al., 1991). Processed samples were stored at −80°C until extraction. Samples were named using the *Deep Rover* Dive number (216 to 230) followed by sample number (e.g., 216S1).

**Table 1:** Description of sample locations and geochemical profiles of mats and pool fluids. Data from Collier et al., 1991.

Sample	Site	Map location	T_max_ (**°**C)^*^	Depth (m)	pH	CO_2_ mM	O_2_ μM	SO_4_ mM	Fe nM	Mn nM	NO_3_ μM
216S1^†^	Brain Mat	Llao’s Bath & Brain Mat Complex	4.54	463	7.13	1.26	nd	0.31	380	3.9	1.17
223S1	Llao’s Bath	Llao’s Bath & Brain Mat Complex	4.52	467	6.27	3.00	228.2	0.25	18	1,680	1.44
226S1	Llao’s Bath	Llao’s Bath & Brain Mat Complex	10.2	448	nd	4.96	nd	0.69	41	12,300	0.59
226S2	Near Llao’s Bath	Llao’s Bath & Brain Mat Complex	10.2	448	7.26	0.69	292.6	0.11	nd	nd	1.91
226S3^‡^	Near Llao’s Bath	Llao’s Bath & Brain Mat Complex	10.2	448	7.59	nd	nd	0.55	30,421	15,272	2.96
230S1	Brain Mat	Llao’s Bath & Brain Mat Complex	5.98	443	7.23	4.95	17.7	0.55	254	25,200	0.61
230S3	Llao’s Bath Milky Pool Mat	Llao’s Bath & Brain Mat Complex	5.98	443	7.14	0.78	279.2	0.15	nd	nd	2.55
228S3	Palisades Point Pool Mat	Palisades Point Pools	9.68	554	7.97	2.70	nd	nd	42	38	1.57
Crater Lake Bottom Water	Composite	Deep Lake	3.6	429	6.95	0.64	295	0.10	0.2	3.0	1.39

* Ambient temperature = 3.6°C.

† Metagenomic analysis.

‡ SSU rRNA clone library analysis.

### DNA extractions

DNA extractions were performed on mat and pool fluid samples using the FastDNA Spin Kit for Soil (MP Biomedicals, Irvine, CA) protocol, which was followed according to the manufacturer’s instructions. Lysis was performed with two rounds of bead beating for 45 s at a setting of 5.5 using the FastPrep instrument with samples being placed on ice between runs. DNA was eluted in 100 μl of 1.0 mm Tris pH 8.0. Total DNA was quantified with a Qubit 2.0 fluorometer using the dsDNA HS kit (Thermo Fisher Scientific, Waltham, MA).

### SSU rRNA gene PCR amplification and clone library analysis

Each sample was then processed within weeks of gDNA extraction, where bacterial SSU rRNA genes were amplified from the gDNA using the 68F forward primer (5’ TdNA dNAC ATG CAA GTC GdKdK CG 3′) and the 1492R reverse primer (5’ dKGdP TAC CTT GTT ACG ACT T 3′), where dK is a purine analog, dP is a pyrimidine analog, and dN is an equal mixture of dK and dP (Glen Research, Sterling, VA). Five replicate PCRs were performed using ~50 ng of gDNA template, 5 U of Taq polymerase, 2.5 mm MgCl_2_, 200 μm each dNTPs, 1 μm (each) forward and reverse primers, and molecular-grade water to a total volume of 50 μl. The following conditions were used for the amplification process: an initial 8-min hot start at 95°C, followed by 25 to 30 cycles of denaturation (94°C for 1 min), annealing (58°C for 90 s), and elongation (72°C for 3 min). This was followed by a final elongation step at 72°C for 7 min. Amplicons were sized by 1% agarose gel electrophoresis against a 1-kb ladder. Negative controls were maintained throughout. The five replicate PCR mixtures were pooled, concentrated, and desalted. The desalted PCR amplicons were then cloned with a Topo-TA cloning kit following the manufacturer’s instructions (Thermo Fisher Scientific). All putative clones were streaked for isolation, and the inserts were assayed for correct size using PCR with M13F and M13R primers prior to sequencing (Moyer, 2001).

### SSU amplicon sequencing and analysis

For all samples, the V3-V4 variable regions of the SSU rRNA gene were amplified *via* polymerase chain reaction (PCR) from all samples using bacterial primers 340F-CCTACGGGNGGCWGCAG and 784R-GGACTACHVGGGTATCTAATCC (Klindworth et al., 2013) with Illumina compatible adaptors. Triplicate PCRs were performed in 25 μl reactions with 2X KAPA HiFi HotStart ReadyMix (Kapa Biosystems, Wilmington, MA), 0.1 mm forward/reverse primers, and 25 ng template DNA. The following PCR conditions were used: 3 min at 95°C; 25 cycles of 30 s at 95°C, 30 s at 55°C, and 30 s at 72°C; a final elongation of 5 min at 72°C; and a hold at 4°C. PCR products were pooled and purified using Agencourt AMPure XP beads (Beckman Coulter, Brea, CA). Adapters with unique index combinations were added to each sample in a 50 μl PCR using 2X KAPA HiFi HotStart ReadyMix with the following conditions: 3 min at 95°C; 8 cycles of 30 s at 95°C, 30 s at 55°C, and 30 s at 72°C; a final elongation of 5 min at 72°C; and a hold at 4°C. Products were again purified with AMPure XP beads. Libraries were quantified with a Qubit 2.0 fluorometer. Sequencing was performed on an Illumina MiSeq generating 2 × 300 bp using paired-end reads.

Amplicon sequence reads were quality checked using FastQC (Andrews, 2010). Amplicons were then processed using the mothur software package (Schloss et al., 2009; Kozich et al., 2013). After forming contigs from the paired-end reads, any sequences shorter than 420 bp, longer than 470 bp, or with ambiguous base calls were eliminated from further processing. Reads were aligned to the SILVA v138.1 SSU reference database. Any sequences with a homopolymer greater than 8 bases were also eliminated. Reads were pre-clustered with the pre-cluster command with a threshold of four-nucleotide differences. Chimeras were removed with UCHIME (Edgar et al., 2011). Sequences were binned into OTUs based on 97% sequence similarity, and OTUs were classified to the genus level using RDP training set v18 (Wang et al., 2007).

OTU bins at the level of 97% sequence similarity as determined with mothur were used in all downstream analyses. Reads were randomly subsampled to the number of reads in the least sequenced sample (54,068 contigs) for calculation of Good’s coverage, Abundance-based Coverage Estimator (ACE) richness (Kim et al., 2017), Chao-1 richness (Chao, 1984), and inverse Simpson diversity (Simpson, 1949) with the summary.single command. Non-metric multidimensional scaling in three dimensions was used to assess beta diversity among samples. Abundant OTUs were determined by selecting OTUs with >1% of the total reads/sample in at least one sample, and OTUs with >2% of the total reads/sample in at least one sample (Supplementary Table 1). Abundant autotrophic OTUs were determined by selecting OTUs with autotrophic metabolic potential that comprised >0.5% of the total reads/sample in at least one sample. The ggplot2 package (Wickham et al., 2016) in R (version 3.6.1) was used to visualize OTU taxon-abundance data. Rarefaction curves were calculated using mothur based on the number of observed OTUs per sample and 1000 iterations, and the ggplot2 package in R was used to visualize these data.

Further analysis of OTUs classified to the genus *Mariprofundus* were further assessed using the program *ZetaHunter* (McAllister et al., 2018). *ZetaHunter* assigns Zetaproteobacteria sequences to canonical Zetaproteobacterial OTUs (Zeta OTUs) in the SILVA v123 SSU reference database. Zetaproteobacteria reads across all eight mat communities with an abundance >10 reads were isolated, identified, and assessed using *ZetaHunter* to further classify these Zeta OTUs (Supplementary Table 2).

### Metagenomic sequencing, assembly, and analysis

For sample 216S1, gDNA was further cleaned and concentrated using an Aurora System (Boreal Genomics, Vancouver, BC) prior to metagenomic sequencing. Libraries were prepared with the Nextera DNA library kit (Illumina, San Diego, CA) for Illumina sequencing with 2 × 300 bp paired-end reads. Sequenced reads were quality checked using FastQC (Andrews, 2010) and quality control was performed with Trimmomatic (Bolger et al., 2014).

The quality filtered metagenomic reads were assembled into contigs using the program MegaHIT (Li et al., 2015). Genes were predicted using the program Prodigal (Hyatt et al., 2010). A homology search for both COG and KEGG was then run on the predicted genes using Diamond (Buchfink et al., 2021). Each ORF was then functionally and taxonomically assigned using SqueezeMeta (Tamames and Puente-Sánchez, 2019). Contigs without any gene predictions were analyzed by BlastX with the Diamond sequence aligner for ORF identification. Gene abundances were calculated using STAMP (Parks et al., 2014), then relative abundance was calculated by taking the raw read counts for each orf, dividing it by the total reads in the sample, and multiplying by 100. Coverage values (bases mapped/ORF length) and normalized RPKM values were calculated using SqueezeMeta pipeline scripts.

The results of the pipeline were imported into R, version 3.6.1 using the SQMtools R package, version 0.7.0 (Puente-Sánchez et al., 2020). This package was then used to visualize the taxonomy of the contigs and plot the functional profile of the genes in the samples with KEGG, COG, and PFAM annotations. The taxa with major autotrophy indicator genes were then extracted and plotted using ggplot2.

## Results

### Location descriptions and site geochemistry

**Figure 1:**
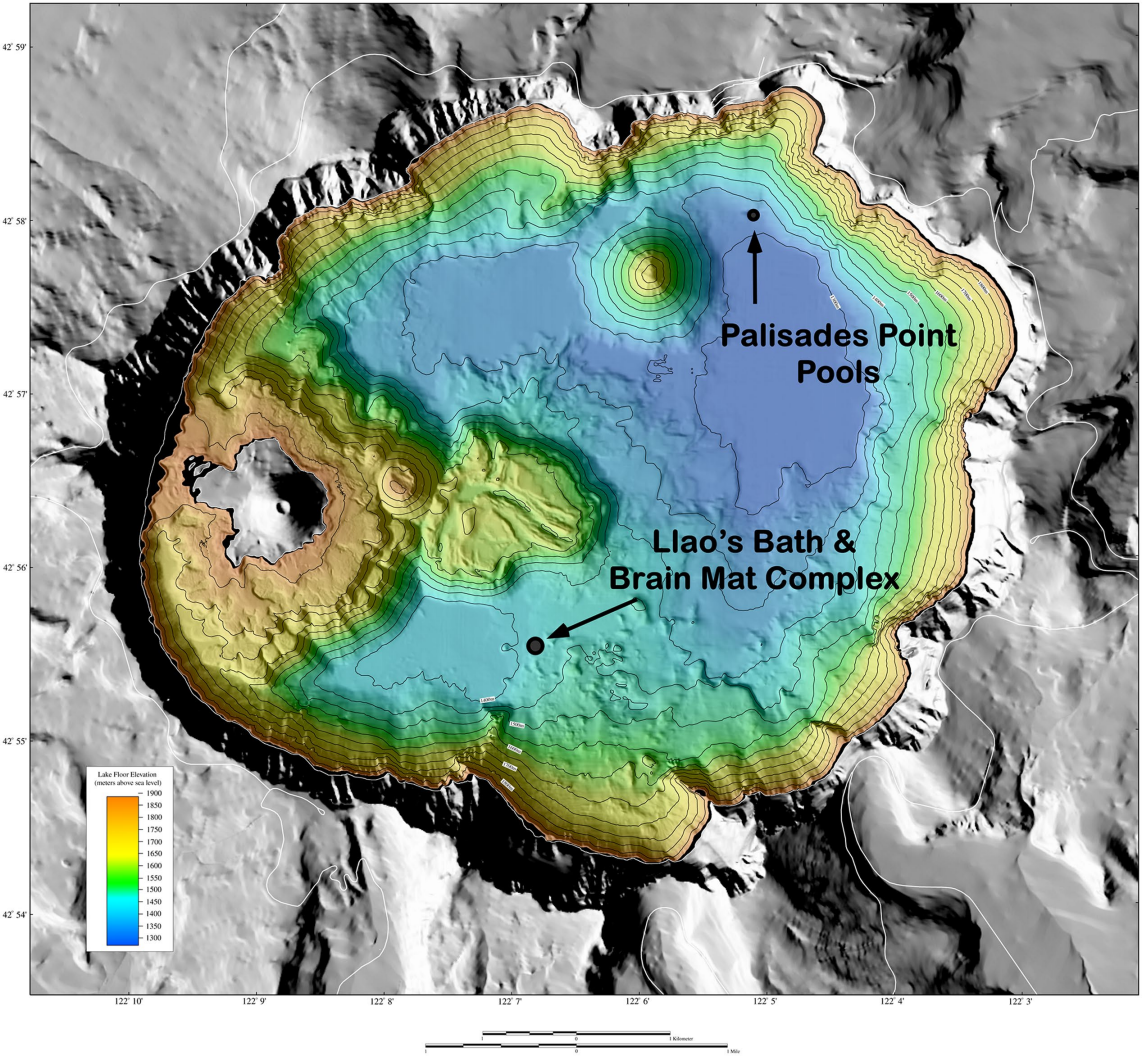
Shaded relief of surrounds and bathymetric map of the floor of Crater Lake, Oregon. Surrounding terrain from USGS 10 m digital elevation model (DEM). DEM illuminated from 225°azimuth, 45°elevation. Colored region is the lake floor (±1 m resolution), whereas the gray region is the surrounding land. The distance across the width of the lake is approximately 9 km (5.6 miles). The reds and yellows show the shallower depths of the lake, whereas the greens and blues show the deeper depths. Adapted from Bacon et al., 2002.

**Figure 2:**
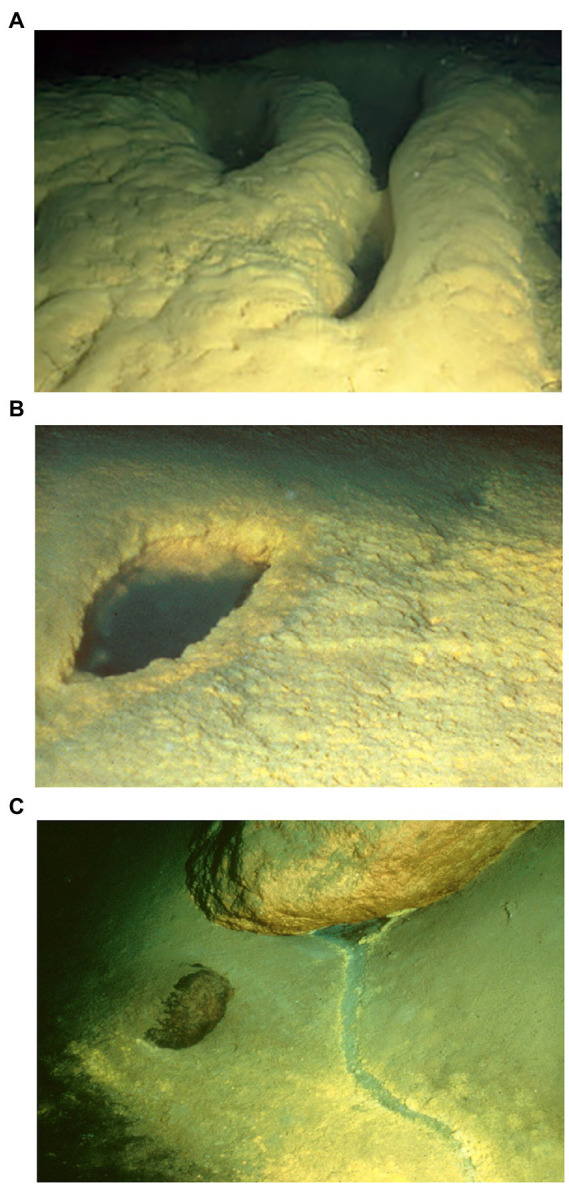
Representative photos of bacterial mat collection sites: **(A)** “Brain mat” bacterial growth west of Llao’s Bath within the Llao’s Bath/Brain Mat complex; **(B)** Llao’s Bath brine pool with surrounding bacterial mat; **(C)** A rill within the Palisades Point area. This channel or rill flows downhill from its origin under the boulder (upper part of the photo). Each photo field of view is approximately three meters across. Images courtesy of Collier et al., 1991.

Llao’s Bath and Brain Mat Complex lies in the south-central area of the lake (Figure 1). It is in an area of low relief and is surrounded by sediment on three sides, while a rounded rocky outcrop projects upward from the northwestern edge of the pool. The pool margin is rimmed with a bacterial mat 10 to 20 cm thick. A gentle slope rises from the western edge of the pool that contains an extensive area also covered by bacterial mats. Due to the convoluted morphology of these mats (Figure 2A), this area was termed the “brain mat” and is spatially associated within a few meters of Llao’s Bath (Figure 2B).

Palisades Point Pools occur in the north-east side of the lake near the terrestrial feature Palisades Point and north-east of the north-central underwater cinder cone Merriam Cone (Figure 1). Many small pools were observed in this area which extends approximately 50 m across by 100 m long and lies in a sedimented area of low relief at the base of the lake’s caldera wall. A unique feature of this pool is the stream-like projections or rills that extend from the upslope region with dendritic patterns that indicate downstream flow (Figure 2C). Bacterial mats line the sides of this pool and occur along the rills much like the mats located along the margins of Llao’s Bath. According to geochemical analyses conducted by Collier et al. (1991), mats and pool fluids were enriched in CO_2_, SO_4_, Fe, Mn, and NO_3_ compared to bulk lake water, which are consistent with fluids of hydrothermal origin (Table 1). While the bulk lake water is near saturation in respect to dissolved oxygen, dissolved oxygen concentrations decreased with depth. This may be impacted by the introduction of anoxic hydrothermal fluids and reduced inorganic ions such as Fe and Mn (Collier et al., 1991). Among sample sites, sample 226S3 from near Llao’s Bath had the highest Fe concentration of 30.4 mm (Table 1). In addition to the notable enrichments in iron, several samples also exhibited elevated Mn concentrations.

### Community structure and diversity

An initial clone library of nearly full-length SSU genes was constructed from sample 226S3 (e.g., from the Llao’s Bath and Brain Mat Complex area) that yielded a total of 76 clones, of these 45 clones were detected at least twice (i.e., not singletons) and comprised 16 operational taxonomic units (OTUs), which were then chosen to be fully sequenced. Of these OTUs, 40% were identified as putative chemoautotrophs, with most of these identified as iron-oxidizing bacteria and were represented by three OTUs belonging to the genus *Gallionella*. Three other OTUs were identified as being represented by the genus *Sulfuricurvum, Nitrosomonas,* and *Nitrospira*, putative sulfur-oxidizing, ammonia-oxidizing, and nitrite-oxidizing bacteria (or possibly complete nitrification), respectively.

Amplicon sequencing was done from seven Llao’s Bath and Brain Mat Complex and one Palisades Point Pool bacterial mat samples. In total 6,646,366 raw, paired-end sequences covering the V3-V4 regions of the SSU rRNA gene were generated. Quality filtering in mothur (Schloss et al., 2009), resulted in 4,167,607 sequences were then analyzed for a more detailed examination of bacterial mat community structure and diversity. At a 97% sequence similarity cutoff, 67,668 OTUs were generated, of which 55 were abundant with >1% of the total reads in at least one sample.

The highest number of observed OTUs, as determined by 97% sequence similarity, occurred in community 226S2 from the Llao’s Bath/Brain Mat Complex (Supplementary Table 1). The 226S2 community had proportionally higher richness and diversity estimates. Rarefaction analysis corroborated these alpha diversity estimates and revealed higher overall diversity in the 226S2 community from Llao’s Bath relative to the remaining seven communities sampled (Supplementary Figure 1). In contrast, rarefaction analysis revealed lower than average overall diversity in the 226S1 and the 226S3 communities, both from the Llao’s Bath area. These findings were corroborated by Inverse Simpson diversity estimates, which revealed the lowest richness and diversity in these two communities (Supplementary Table 1).

**Figure 3:**
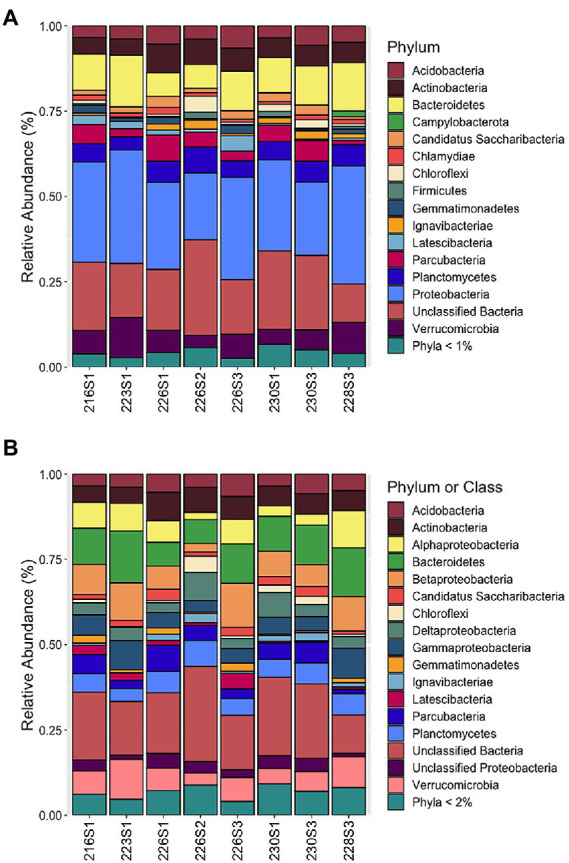
Community structure of eight benthic microbial mats from hydrothermal springs in Crater Lake, Oregon, at various taxonomic levels: **(A)** Community structure of abundant taxa representing >1% total reads per sample in at least one sample at the phylum level. Phyla <1% included as separate taxon; **(B)** Community structure of abundant taxa representing >2% total reads per sample in at least one sample at the phylum level with Proteobacteria shown at the class level.

Although many samples had elevated Fe and Mn concentrations, the bacterial community structure was revealed to be similar across all eight samples that were examined. Non-metric multidimensional scaling in three dimensions revealed even distributions among all communities with no obvious clustering, indicating a low differential in beta diversity (data not shown). The majority of reads from all communities were from the Proteobacteria (NB, recently reclassified as the Pseudomonadota phylum; Oren and Garrity, 2021), at the phylum level (Figure 3A). The Proteobacteria phylum was dominated at the class level by the Betaproteobacteria, Alphaproteobacteria, Gammaproteobacteria, and Deltaproteobacteria (Figure 3B; NB, recently reclassified into four novel phylum-level lineages; Waite et al., 2020). In addition, a large proportion of reads were in the category of unclassified Bacteria with an average and standard deviation across all samples of 19.2%. Other abundant phyla include *Acidobacteria*, *Actinobacteria*, *Bacteroidetes*, *Candidatus Saccharibacteria*, *Chloroflexi, Gemmatimonadetes, Ignavibacteriae, Latescibacteria*, *Parcubacteria*, *Planctomycetes*, and *Verrucomicrobia*.

Betaproteobacteria represented the dominant Proteobacteria class with a maximum value of 13.0% of the community 226S3 from near Llao’s Bath, which also exhibited the highest measured iron concentrations (Table 1). The majority of the Betaproteobacteria were from the genera *Gallionella* as well as *Rhodoferax, Undibacterium*, and an unclassified genus. Next in abundance were the Alphaproteobacteria that had a maximum relative abundance of 10.9% of the Palisades Point Pools community, 228S3. The Alphaproteobacteria were dominated by the genera *Novosphingobium* and *Rhodoferax.* The maximum relative abundance of Gammaproteobacteria also occurred at Palisades Point Pools, comprising 8.8% of the community. The majority of these belonged to the genera *Thiobacillus* as well as *Xanthomonadaceae, Alteromonadales, Silanimonas,* and an unclassified genus. Deltaproteobacteria were the next most abundant class represented by the genera *Geobacter*, *Desulfobacteraceae*, and an unclassified genus with a maximum value of 8.2% from 226S2 near Llao’s Bath. This unclassified Deltaproteobacteria genus represented the most abundant OTU across all eight communities with an average relative abundance of 1.9%.

Variability in community structure was revealed within the less abundant Proteobacteria classes Zetaproteobacteria and Epsilonproteobacteria (NB, recently reclassified as the Campylobacterota phylum; Waite et al., 2017). Community 228S3 from Palisades Point Pools contained the highest relative abundance of Campylobacterota at 1.6% (Figure 3A). This abundance was greater than the average relative abundance for Campylobacterota of 0.06% for the other seven communities. The majority of these were identified from the genus *Sulfuricurvum* that comprised 1.4% of the 228S3 community. Community 226S1 from Llao’s Bath contained the highest relative abundance of Zetaproteobacteria, represented by the single genus *Mariprofundus,* at 0.53% compared to an average relative abundance of 0.02% for the remaining seven communities (Figure 3B).

### Autotroph diversity

**Figure 4:**
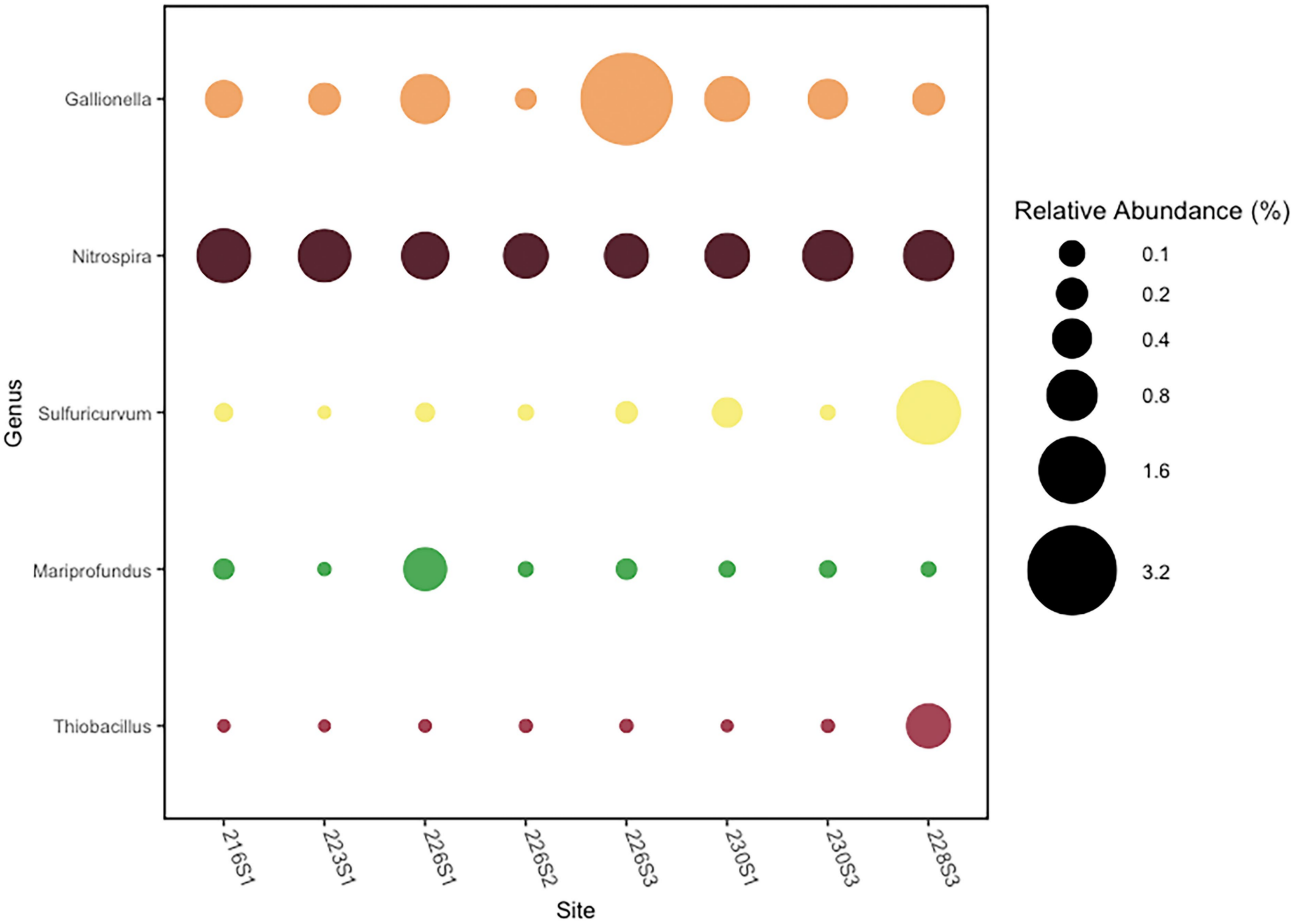
Relative abundance of putative chemoautotrophic genera with a maximum abundance >0.5% of reads in at least one sample.

Putative autotrophic taxa were characterized by OTUs belonging to five genera comprising >0.5% total reads per community that were detected in at least one community (Figure 4). The genus *Gallionella* was the most abundant putatively autotrophic taxa across all eight communities. Community 226S3 collected near Llao’s Bath contained the highest abundance of reads from two *Gallionella* OTUs comprising a combined relative abundance of 3.4% of the total bacterial community. Across all eight communities, the average relative abundance of *Gallionella was* 0.75% of the bacterial community. The next most abundant autotrophic taxon was represented by the genus *Nitrospira,* which had a more consistent distribution across all eight communities, comprising an average relative abundance of 0.73%. The maximum relative abundance of *Nitrospira* occurred in the 216S1 community from Brain Mat comprising 1.0% of the total. Community 228S3 from Palisades Point Pools contained the highest abundance of an autotrophic *Sulfuricurvum* OTU. *Sulfuricurvum* was present in a relative abundance of 1.4% in the Palisades Point Pools community compared with an average relative abundance of 0.04% in the other seven communities. The genus *Mariprofundu*s represented the fourth most abundant autotrophic taxa detected in the Crater Lake bacterial communities. *Mariprofundus* comprised an average relative abundance of 0.08% across all eight communities, with a maximum abundance of 0.53% found in community 226S1 from Llao’s Bath. Finally, the autotrophic genus *Thiobacillus* comprised an average relative abundance of 0.07% of the eight bacterial communities. The maximum relative abundance of *Thiobacillus* occurred in the Palisades Point Pools community at 0.56% (Figure 4).

Sequence reads determined as representing the genus *Mariprofundus* were further processed using the program *ZetaHunter* to provide finer scale OTU classification (McAllister et al., 2018). *ZetaHunter* assigned reads to eight previously characterized Zeta OTUs and one newly described New Zeta OTU 1; however, 452 reads (~0.01%) were unable to be classified representing novel Zetaproteobacteria phylotypes (Supplementary Table 2). The majority of reads were assigned to either Zeta OTU 2 or Zeta OTU 6. The average relative abundance of Zetaproteobacteria in the Crater Lake bacterial communities was ~0.08%. Zeta OTU 2 and Zeta OTU 6 each comprised a relative abundance of ~0.02% of the pooled Crater Lake community, respectively.

### Metabolic potential of the Crater Lake bacterial mat community

The metagenomic sequencing of sample 216S1 resulted in 26,096,482 total reads that were assembled into 319,555 contigs. From these contigs, there were 616 SSU rRNA genes identified. Of which, 196 were taxonomically assigned; one-third of those being unclassified Bacteria. The next most abundant taxa were Betaproteobacteria and Planctomycetes, with seven contigs assigned to each class. Of the top 15 most abundant KEGG functional genes assigned to contigs, six were involved in cellular transport. The most abundant KEGG functional gene was determined to be a serine/threonine protein kinase with approximately 800 reads per million total reads (Supplementary Figure 2).

**Figure 5:**
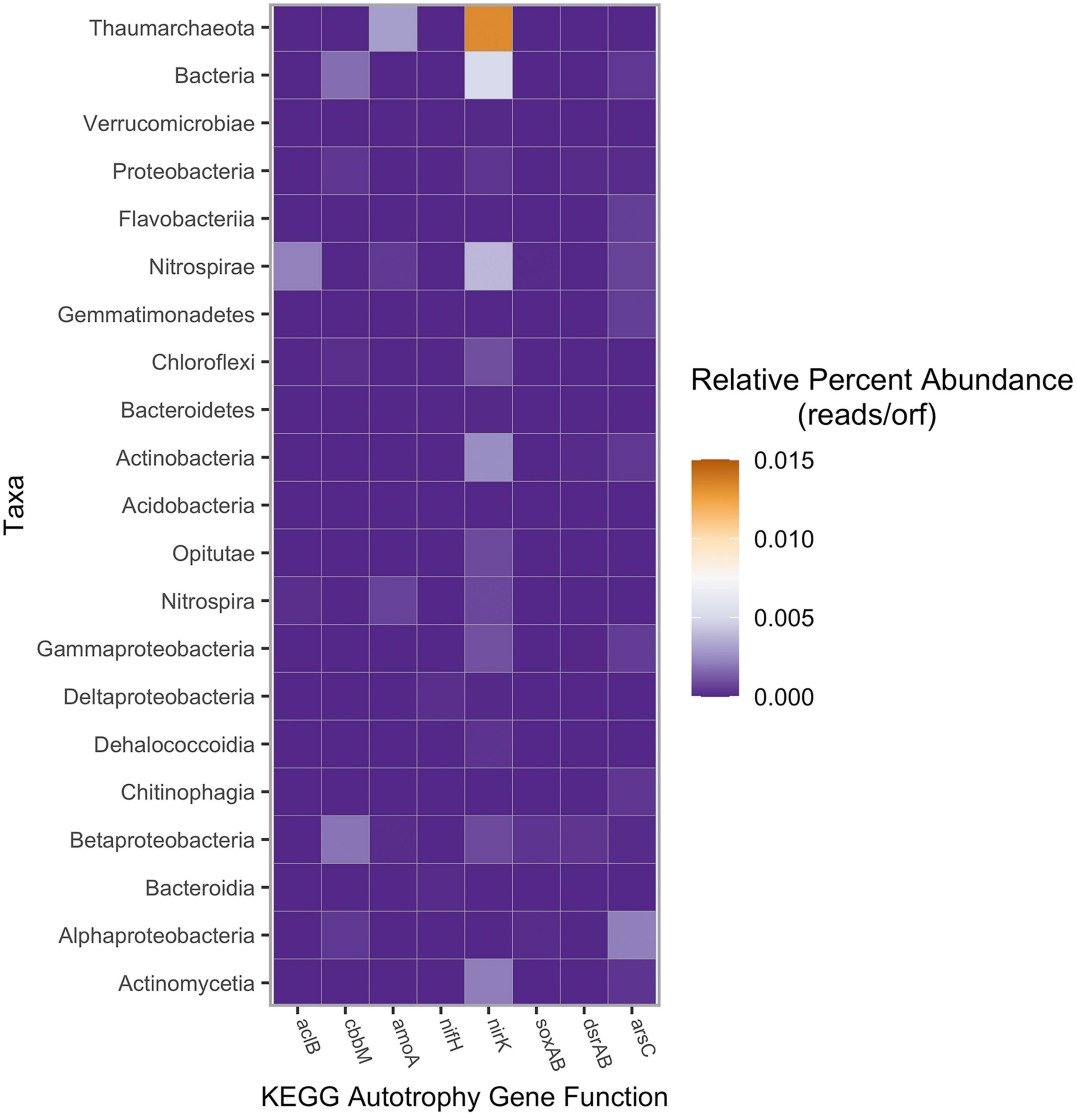
Autotrophy gene abundance based on assigned taxa, measured in percent of raw reads per total number of reads in the sample. KEGG gene abundance was calculated using SqueezeMeta with a lowest abundance threshold of 1.25 × 10^−5^ reads per ORF (Tamames and Puente-Sánchez, 2019). Taxa represented by super-taxa (e.g., Thaumarchaeota, Bacteria, etc.) were each the unclassified residual reads not included in the sub-taxa (either phyla or class) listed below, respectively.

To better understand nutrient cycling and primary productivity, the abundance of specific genes was further investigated (Figure 5). Two genes of interest were *aclB* and *cbbM*, which are key for the reductive TCA cycle and the reductive pentose phosphate cycle, respectively. Within this microbial mat, genes encoding *cbbM* were more abundant than *aclB*, suggesting primary production *via* reductive pentose phosphate cycle is the dominant autotrophic pathway. The high oxygen Form I of RubisCO was not identified within this metagenome.

Additionally, abundance of genes for the nitrogen and sulfur cycle was analyzed. Key genes of the nitrogen cycle, *amoA* (ammonia monooxygenase), *nirK* (nitrite reductase), and *nifH* (nitrogenase) had variable abundances. Ammonia monooxygenase was identified in the bacterial phylum Nitrospirota and in the class Nitrospira. However, *amoA* was also detected in the archaeal unclassified Thaumarchaeota at relatively high abundances. Nitrite reductase genes were identified across most of the major classes of autotrophic organisms and in the archaeal class Nitrososphaeria, whereas nitrogenase was only identified in three taxa at minimal levels. Genes involved in the sulfur cycle were more taxonomically restricted in comparison to nitrite reductase. The Betaproteobacteria contained genes encoding *dsrAB* and *soxAB*, suggesting both dissimilatory sulfate reduction and sulfur oxidation were important in respiratory pathways. Additionally, both the oxidative and reductive forms of *dsrAB* were found to be present. Betaproteobacteria, a taxon found to be most prevalent with SSU rRNA analysis, had nearly all the investigated functional genes except for *aclB* and *nifH*. Arsenic detoxification gene *arsC* (arsenate reductase) was identified in only a few taxa and was most abundant within the Alphaproteobacteria (Figure 5).

## Discussion

### A generally homogeneous bacterial community

Compared to other hydrothermal vent fields, the bacterial mats found in the basin of Crater Lake generally represent an unexpectedly homogeneous overarching community with a relatively large number of observed OTUs and a community structure characterized by relatively high species richness and alpha diversity. The low beta diversity and low spatial variability among sites were surprising given the highly dynamic and variable nature of microbial communities at other hydrothermal sites, even communities in extremely close proximity (Davis and Moyer, 2008; Sheik et al., 2015). In addition, the high alpha diversity observed in the mats is surprising given the extreme oligotrophic nature of Crater Lake that often supports lower microbial diversity when compared to eutrophic lake systems (Newton and McLellan, 2015). This observed low spatial variability starkly contrasts with other hydrothermal vent ecosystems where heterogeneous vent effluent and episodic vent fluid composition, temperature, and flow rate results in distinctly different mat communities across small spatial scales (Nakagawa and Takai, 2008). For example, in the Mariana Arc and back-arc systems, hydrothermal vent microbial community structure has been shown to be extremely diverse even within a vent field (Emerson and Moyer, 2010). Another example of iron-dominated heterogeneous vent effluent leading to high bacterial community variability within a vent field occurs at Lōʻihi Seamount (Duchinski et al., 2019). The high beta diversity within these vent sites is linked to heterogeneous vent effluent providing variable geochemical species for microbial metabolic use (Hager et al., 2017); however, like Crater Lake springs, these vent fluids are enriched in reduced sulfur and iron compounds. The conditions at deep-sea hydrothermal vents are most likely distinctly different from the Crater Lake benthic microbial mats, where more variable hydrothermal fluid flow combined with higher heat flux results in geochemistry that is more temporally and spatially heterogeneous.

Selected diversity metrics from the Mariana Arc and back-arc and Lōʻihi Seamount hydrothermal ecosystems highlight the high alpha diversity and low beta diversity observed among Crater Lake bacterial mat communities (Supplementary Table 3). The high OTU richness (Chao 1 richness) and OTU evenness (Inverse Simpson diversity) at all eight sites is surprising given the extremely oligotrophic nature of Crater Lake, restricting diverse metabolic potential and ecological niches. In additional to diversity measures, the low beta diversity found among the mats can also be demonstrated by comparing abundant OTUs. Of the 55 dominant OTUs comprising >1% of the total reads in at least one sample, 54 OTUs were cosmopolitan across all eight sites. In contrast, across 22 bacterial mat communities from four vent fields in the Mariana Arc and back-arc, 162 OTUs were abundant with >1% of the total reads in at least one sample. Of these 162 OTUs, only 30 were cosmopolitan taxa across all mats (Hager et al., 2017). The ubiquitous and abundant OTUs detected across all eight sites indicate an exceptionally homogeneous bacterial mat community spanning the hydrothermal springs of Crater Lake. It is possible that the lower diversity observed in mat communities 226S1 and 226S3 revealed by rarefaction analysis (Supplementary Figure 1) may be attributed to these samples being taken from older, lower biomass mats instead of being from distinct, less diverse communities.

### Gallionella

Among the taxa identifiable to genus level by both clone library and amplicon sequencing analysis, *Gallionella* were found to be the most abundant autotrophic genus within the Crater Lake bacterial mat community. However, there were many unclassified groups within the community that may also have contributed to mat primary production. The putative iron and manganese-oxidizing *Gallionella* produce twisted sheaths of precipitated iron hydroxides because of their metabolic processes that likely form the bacterial mat matrices at Crater Lake hydrothermal springs (Figure 2). *Gallionella* comprised an average relative abundance of 0.75% of the mat communities. A maximum relative abundance of 3.4% occurred at the site containing the highest concentration of iron, 226S3, indicating that this bacterial mat community is likely driven by iron oxidation by *Gallionella* over sulfur oxidation. In addition, high observed concentrations of manganese in all mat sites except 216S1 further support the conclusion that these iron and manganese-rich mats are driven by autotrophic *Gallionella.* These data corroborate the findings of Dymond et al. (1989), that *Gallionella* comprise a dominant proportion of the Crater Lake bacterial mat communities. However, no bacteria from the genus *Leptothrix* were detected in this study. *Leptothrix* growth is co-limited by calcium and magnesium; therefore, it is probable that the concentrations of these minerals were too high to support the growth of *Leptothrix* (Eggerichs et al., 2020). Zetaproteobacteria is another sheath-forming iron oxidizer (Emerson et al., 2007) that may have been misidentified as *Leptothrix*.

### Zetaproteobacteria

The genus *Mariprofundus* from the class Zetaproteobacteria represented a small proportion of the Crater Lake bacterial mats with an average of 0.08% of the community but functions as an iron oxidizer that may contribute to the matrix of these mats (Emerson and Moyer, 2002; McAllister et al., 2011). The relative abundance of Zetaproteobacteria ranged from a minimum of 0.001% in sample 223S1 to a maximum of 0.53% in sample 226S1. The Crater Lake metagenome was constructed from sample 216S1, which had 0.04% of Zetaproteobacteria; however, no evidence of carbon fixation by Zetaproteobacteria was identified, which could be attributed to their low relative abundance.

Zetaproteobacteria are typically found in marine or brackish iron-rich environments and have only recently been found in terrestrial and coastal ecosystems (McAllister et al., 2019); therefore, detecting Zetaproteobacteria in the ultra-oligotrophic freshwater system at Crater Lake is a novel finding that expands their observed range. In addition, finding Zetaproteobacteria in a community with abundant *Gallionella* is also noteworthy. A highly significant correlation between relative abundances of *Mariprofundus* and *Gallionella* has been observed in marine hydrothermal iron mat communities (Vander Roost et al., 2017); therefore, finding these two taxa co-occurring in benthic mats at Crater Lake provides further evidence of their co-colonization of hydrothermal iron mats.

Fine-scale characterization of Zetaproteobacteria was conducted by using *ZetaHunter*, which assigns Zetaproteobacterial reads to canonical Zeta OTUs. The majority of Zetaproteobacteria detected in the Crater Lake community were assigned to either Zeta OTU 2 or Zeta OTU 6 (Supplementary Table 2). Zeta OTU 2 has been shown to be globally distributed and is often the most abundant Zeta OTU found in iron-dominated hydrothermal systems (McAllister et al., 2011; Duchinski et al., 2019). Zeta OTU 6 has also been found in lower abundances in hydrothermal iron mats although its distribution is generally more prevalent in coastal sediments and mineral weathering incubations. The discovery of Zetaproteobacteria at Crater Lake further expands the role of iron-oxidizing Zetaproteobacteria in freshwater iron-rich ecosystems and contributes to the understanding of Zetaproteobacteria habitat and niche preference.

### Other notable autotrophs

In addition to iron oxidization by *Gallionella* and *Mariprofundus*, other reduced inorganic compounds may provide key nutrients to autotrophic taxa. The second most abundant autotrophic taxon found across all eight bacterial communities is from the genus *Nitrospira* belonging to the phylum Nitrospirota. The metagenomic data indicates that the Crater Lake *Nitrospira* have genes for nitrite reductase, indicating their potential role in denitrification. Because nitrite can be fatal to fish and other vertebrates, the presence of denitrification by *Nitrospira* is critically important in marine and freshwater ecosystems. Some members of the genus *Nitrospira* are capable of complete nitrification by oxidizing ammonia and nitrite into nitrate; these organisms are known as complete ammonia oxidizers or “comammox.” The low allochthonous input, highly oligotrophic conditions present in Crater Lake, and the aggregation of benthic bacterial communities into microbial mats suggest that these abundant *Nitrospira* may be comammox (Daims et al., 2015). It has also recently been shown that when Fe(II) and nitrate are present in freshwater oligotrophic ecosystems that the presence of *Gallionella* enhances nitrate-reducing microbial assemblages (Jakus et al., 2021).

Another putative autotrophic sulfur-oxidizer within the mat community was represented by the genus *Thiobacillus*. The highest abundance of *Thiobacillus* comprising 0.56% of the bacterial community occurred at Palisades Point Pool, providing further evidence that this community is driven by the inclusion of sulfur oxidation as a source of primary production. One member of this genus, *Thiobacillus denitrificans*, has been shown to oxidize ferrous sulfide in the presence of nitrate (Straub et al., 1996; Hedrich et al., 2011). The detection of *Thiobacillus* and *Nitrospira*, particularly in the higher relative abundances observed in the Palisades Point Pool community, may provide the support that comammox *Nitrospira* are producing nitrate and driving ferrous sulfide oxidation by *Thiobacillus.* In addition, metagenomic analysis reveals the highest abundance of genes involved in sulfur oxidation, *soxAB*, belong to the Betaproteobacteria, providing further support that sulfur oxidation by *Thiobacillus* could be a significant source of primary production in Crater Lake mat communities. A relatively high abundance of Campylobacterota (formerly known as Epsilonproteobacteria) dominated by the genus *Sulfuricurvum* was also observed across all eight bacterial mats and was also most highly enriched at Palisades Point Pool comprising ~1.4% of the community. *Sulfuricurvum* spp. are chemolithoautotrophs which oxidize sulfur and fix carbon *via* the rTCA cycle, which allows them to be early colonizers of hydrothermal systems (Handley et al., 2014; Waite et al., 2017). The elevated relative abundance of *Sulfuricurvum* present in community 228S3 provides additional support that Palisades Point Pool mat community is the most reliant on sulfur of all the communities that were investigated. However, in the metagenomic sample, no *aclB* genes were associated with the Campylobacterota.

### Sulfur and iron fuel dominant autotrophs

The spring-derived fluids showed elevated levels of CO_2_, iron, and manganese and low levels of oxygen, which support the chemosynthetic microbial mat. The putative autotrophic taxa identified in notable abundances >0.5% of the total mat community in at least one community reveal the importance of iron and sulfur to the microbial mats. Four out of five abundant autotrophic taxa are chemoautotrophs that can utilize iron or sulfur oxidation reactions for energy, while the fifth taxon can use nitrite oxidation. Abundant autotroph diversity is largely homogeneous with respect to spatial distribution, yet slight variability in autotroph diversity may be attributed to slight variability in observed geochemistry among the mat sites. Previous work has suggested the importance of reduced iron as an energy source to these benthic mat communities; however, this is the first time that the contribution of reduced sulfur to the energy demand of the Crater Lake microbial mats has been suggested. This is also especially notable as normally sulfur-oxidizing bacteria occur at higher *in situ* temperatures, as the maximum temperature differential measured across these microbial communities reaches only just over 6°C above ambient.

The presence of autotrophy genes within the metagenome of the Crater Lake microbial mat confirms the prevalence of chemoautotrophs. Not only were similar taxa found to have these genes indicative of autotrophy, but these genes were present in relatively high abundance. Additionally, the most abundant taxa from the metagenomic analysis were found to have genes for autotrophic lifestyles. The identification of the form II RubisCO to the exclusion of form I indicates adaptation to low oxygen concentrations (Tabita et al., 2008; Böhnke and Perner, 2017). Crater Lake bottom water had elevated levels of oxygen in comparison to what was measured at the microbial mats (Table 1).

Further evidence for the importance of sulfur oxidation to the productivity of the Crater Lake mat community is seen in the abundance of both the dissimilatory sulfur reductase (*dsrAB*) and sulfur oxidase (*soxAB*). The most abundant gene involved in sulfur metabolism is the dissimilatory sulfur reductase gene *dsrAB*, followed closely by the sulfur oxidation gene *soxAB*, both identified in the highest abundances as Betaproteobacteria. The co-occurrence of *dsrAB* and *soxAB* genes in Betaproteobacteria is likely due to these sulfur-oxidizing autotrophs utilizing both sets of genes in the oxidative direction; some sulfur-oxidizing bacteria have been found to express both *dsrAB* and sox*AB* genes (Müller et al., 2015; Watanabe et al., 2019). In the active crater volcano lake El Chichón, genes involved in oxidative and reductive sulfur metabolisms were found in high abundances in sediment microbial communities, indicating that sulfur cycling is critical in other crater lake benthos (Peña-Ocaña et al., 2022).

In addition to the Betaproteobacteria, other taxa were found to contain genes for sulfur metabolism. Another taxon with abundant *dsrA* is the Actinobacteria. Actinobacteria are one of the few microbial taxa with the capacity to reduce sulfite to sulfide using the dissimilatory sulfite reductase pathway (Anantharaman et al., 2018). Other taxa identified with abundant sulfur metabolizing genes include Gammaproteobacteria and Acidobacteria (*dsrB*), Alphaproteobacteria (*soxA*, *soxB*), and Nitrospirota (*soxB*). Detecting sulfur-oxidizing *soxB* genes in Nitrospirota may indicate that these organisms are oxidizing reduced sulfur compounds to fuel nitrite reduction. Previous studies have shown sulfate concentrations below 0.4 mm in freshwater oligotrophic lakes (Holmer and Storkholm, 2001). Samples from Crater Lake range from 0.11 to 0.69 mm. At Picard and Von Damm Vents, both of which have higher temperature fluids than Crater Lake, *dsrAB* and *soxA* were detected in metagenomes and metatranscriptomes (Galambos et al., 2019). At the low-temperature vents of Lōʻihi Seamount, sulfide is not abundant in the vent fluids (Glazer and Rouzel, 2009); however, many sulfur-oxidizing bacteria have been identified in the microbial mats (Fullerton et al., 2017). Therefore, it is likely these elevated sulfate values are the result of sulfur oxidation as indicated by metagenomic analyses in comparison to other well-studied hydrothermal vents.

In addition to sulfur cycling, genes for nitrogen cycling were identified to be abundant within the putative autotrophs. Nitrate concentrations ranged from 0.6 to 3.0 μm, a majority of which were elevated in comparison to Crater Lake bottom water. Other hydrothermal vents show low levels of *nifH*, similar to what is seen in the Crater Lake metagenome (Jesser et al., 2015; Anantharaman et al., 2016). Surprisingly, the two most abundant taxa with *nirK* and *amoA* were identified to be archaea. However, other vent systems have identified Thaumarchaeota *amoA* as abundant, such as in the hydrothermal vent plume of Guaymas Basin (Lesniewski et al., 2012). From the Crater Lake metagenome, less than 2% of reads were identified as archaeal, which is in line with other iron-dominated and low-temperature hydrothermal vent systems, such as Lōʻihi Seamount (Davis and Moyer, 2008). However, within the metagenome, the Thaumarchaeota showed relatively high abundance for these two genes. It is unknown if these archaeal taxa are contributing to primary production, since no genes from the 3-hydroxypropionate/4-hydroxybutyrate cycle were identified (Hügler and Sievert, 2011).

## Conclusion

This study reveals the microbial biodiversity of hydrothermal microbial mat communities at the bottom of Crater Lake, Oregon. SSU rRNA gene amplicon sequencing from eight bacterial mats revealed a relatively homogeneous, yet diverse mat community. High alpha diversity in terms of richness and low beta diversity or variability among bacterial mats indicates that these communities are likely fueled by more homogeneous hydrothermal fluids than might have been predicted by the instantaneously collected, microbially relevant geochemical data. The examination of autotrophic taxa abundance revealed the potential importance of iron and sulfur inputs from benthic hydrothermal springs to the primary productivity of these mats. This is further supported by metagenomic analysis showing a potential for carbon fixation by both the rTCA and RPP cycles. Chemoautotrophic potential within the mats was dominated by iron oxidation from *Gallionella* and *Mariprofundus* and by sulfur oxidation from *Sulfuricurvum* and *Thiobacillus* with an additional contribution of nitrite oxidation from *Nitrospira*. These bacterial mat community structure data link the importance of the detected chemoautotrophic metabolisms driven by fluids derived from benthic hydrothermal springs to Crater Lake’s entire lentic ecosystem.

## Data availability statement

The datasets presented in this study can be found in online repositories. The names of the repository/repositories and accession number(s) can be found at: https://www.ncbi.nlm.nih.gov/genbank/, OM194204 through OM194219 https://www.ncbi.nlm.nih.gov/, SRA BioProject PRJNA792592.

## Author contributions

CM and HF conceived, designed, and supervised the analysis of the experiments. AS, LM, HF, and CM wrote the manuscript. AS conducted SSU amplicon sequencing analysis. LM conducted metagenomic sequence analysis. All authors contributed to the article and approved the submitted version.

## Funding

This work was funded in part by Western Washington University’s Office of Research and Sponsored Programs to CM, AS, and LM and by the Biology Alumni Student Research Fellowship to AS and LM, and by the Fraser Family Endowment summer research fund to AS. Funds were also provided by the Fouts Foundation for the Enhancement of Student Research Experiences to CM and the College of Charleston Department of Biology Research and Development fund to HF.

## Conflict of interest

The authors declare that the research was conducted in the absence of any commercial or financial relationships that could be construed as a potential conflict of interest.

## Publisher’s note

All claims expressed in this article are solely those of the authors and do not necessarily represent those of their affiliated organizations, or those of the publisher, the editors and the reviewers. Any product that may be evaluated in this article, or claim that may be made by its manufacturer, is not guaranteed or endorsed by the publisher.
