# Supplementary material for: Unexpected diversity found within benthic microbial mats at hydrothermal springs in Crater Lake, Oregon
Source: Front Microbiol. 2022 Sep 14;13:876044. doi: 10.3389/fmicb.2022.876044 (PMC9516098; doi:10.3389/fmicb.2022.876044)
Supplement: Supplementary file 1 [file Data_Sheet_1.pdf]

Supplemental Table 1. Observed Operational Taxonomic Units (OTUs) with mat community associated diversity metrics. †

Sample	Site	Observed OTUs	Good's coverage	ACE richness	Chao-1 richness	Inverse Simpson diversity
216S1	Brain Mat	5342	0.95	10180	10138	222
223S1	Llao's Bath	4870	0.96	9238	8914	227
226S1	Llao's Bath	3862	0.96	7378	7293	155
226S2	Near Llao's Bath	6419	0.94	11750	11714	337
226S3	Near Llao's Bath	3257	0.98	4996	4708	89
230S1	Brain Mat	5037	0.97	6399	6133	332
230S3	Llao's Bath Milky Pool Mat	5322	0.95	10632	10632	207
228S3	Palisades Point Pool Mat	4925	0.96	8947	8664	219

†All metrics calculated with subsampling to the lowest total number of reads per sample dataset: 52,068.

Supplemental Table 2. Zetaproteobacteria phylotype and relative abundance for all samples representing the total Crater Lake microbial mat community. All Zetaproteobacteria sequences used represent greater than 10 reads per the total Crater Lake mat community. Zetaproteobacteria phylotype determined by *ZetaHunter* (McAllister *et al.*, 2018).

Zeta OTU	Total reads in Crater Lake microbial mat community	Relative abundance in Crater Lake mat community (% x 10^2)†
Zeta OTU 1	105	0.283
Zeta OTU 2	726	**1.956**
Zeta OTU 4	76	0.205
Zeta OTU 6	694	**1.870**
Zeta OTU 7	211	0.569
Zeta OTU 10	24	0.065
Zeta OTU 13	17	0.046
Zeta OTU 35	43	0.116
New Zeta OTU 1	15	0.040
Unclassified Zetas	452	**1.218**
All Zetaproteobacteria	2363	**6.367**

† >1 or >0.01 % in bold.

Supplemental Table 3. Diversity metrics of Lōʻihi Seamount and Mariana Arc and back-arc. Adapted from Duchinski *et al.*, 2019. †

Sample	Vent field	Location	Observed OTUs	Chao-1 richness	Inverse Simpson diversity
672BM1B12345	Lōʻihi	Hiolo North	4604	7889	14.8
674BM1A2356	Lōʻihi	Hiolo North	2767	5930	14.4
674BM1B123	Lōʻihi	Hiolo North	3927	9013	11.8
674BM2C126	Lōʻihi	Pohaku	2143	4821	4.5
674BM2C345	Lōʻihi	Pohaku	1784	4998	9.5
674BM2D12456	Lōʻihi	Pohaku	1622	4713	1.4
675BM1A456	Lōʻihi	Hiolo South	1092	2140	11.9
675BM2A456	Lōʻihi	Hiolo South	2130	4283	21.7
676BM1C34	Lōʻihi	Hiolo North	2461	5658	16.8
676BM2A5	Lōʻihi	Caldera	3879	9383	57.1
797B12	Mariana	Snap Snap	3456	7755	36.8
797B3	Mariana	Snap Snap	4816	11407	14.5
797B56	Mariana	Snap Snap	4130	8387	42.2
797C34	Mariana	Saipanda Horn	2558	4490	28.8
798B123456	Mariana	Champagne	1850	6653	3.3
800B12456	Mariana	Olde Iron Slides	7247	12567	27.2
801X126	Mariana	Golden Horn Base	2607	4952	15.4
801X345	Mariana	Golden Horn Middle	3015	8064	18.5

†All metrics calculated with subsampling to the lowest sequencing dataset.

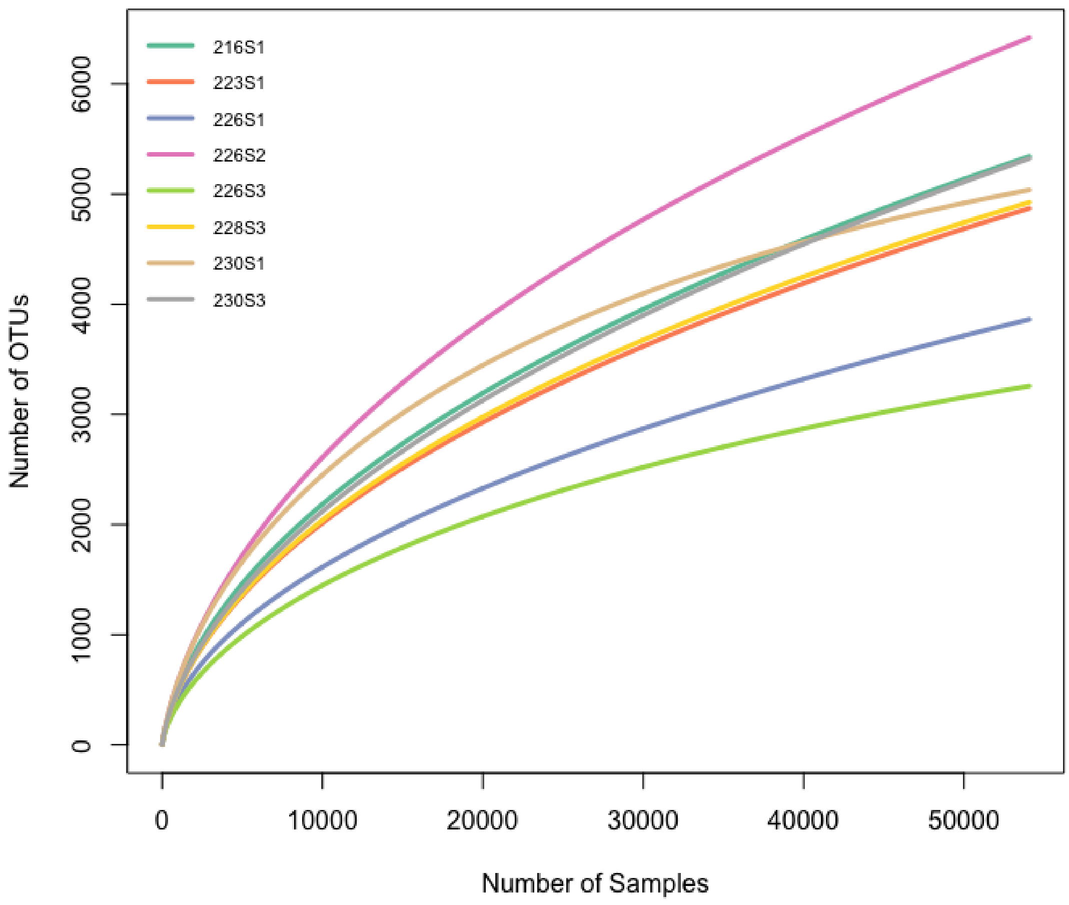

Supplemental Figure 1. Rarefaction curves for Crater Lake Bacterial OTUs based on 97% sequence similarity criterion for eight microbial mat communities.

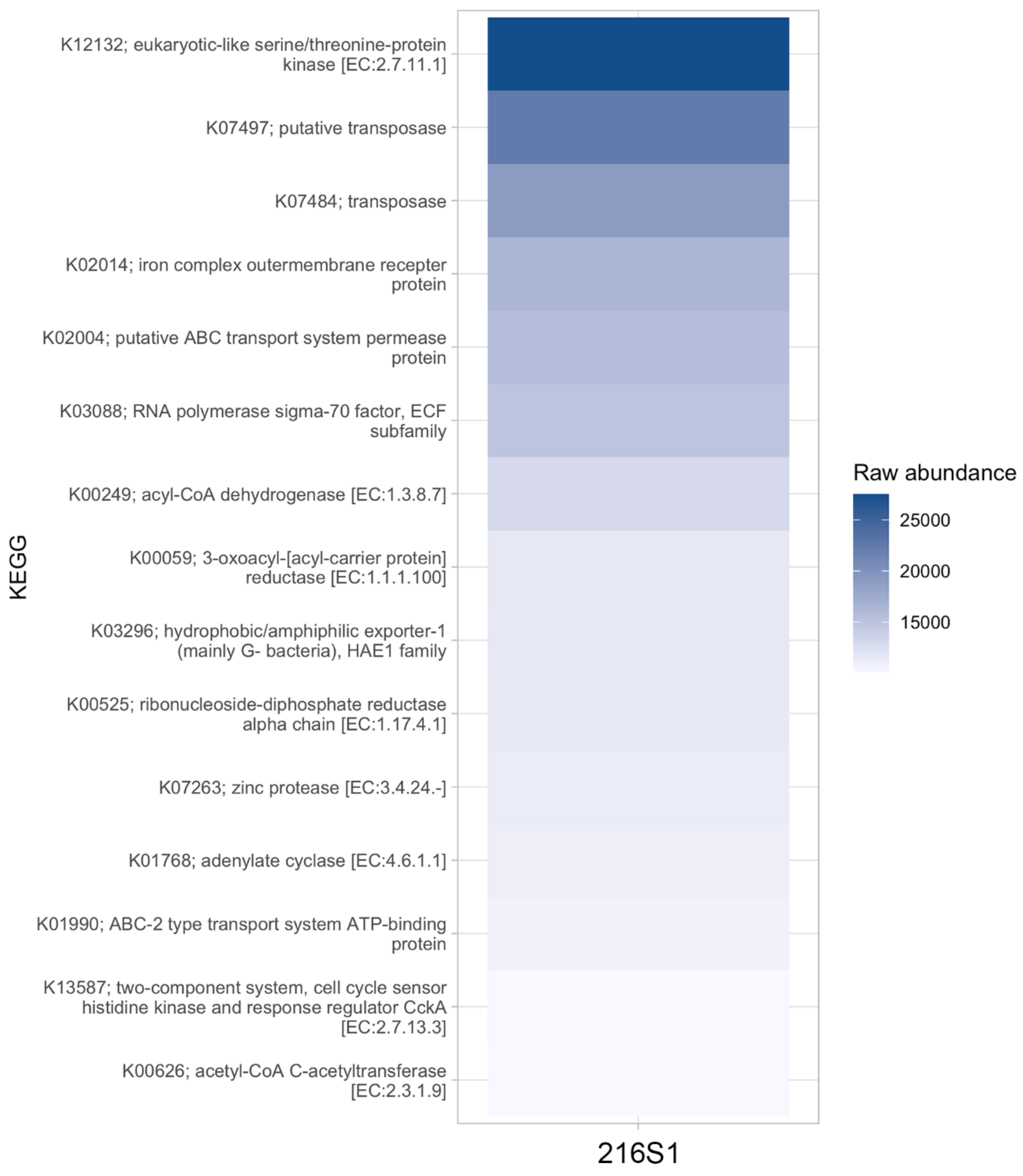

Supplemental Figure 2. The top 15 functionally annotated KEGG genes present in metagenome from Crater Lake mat sample 216S1. Abundance is measured in raw reads. Genes are annotated using Diamond (Buchfink *et al.*, 2021). Abundance calculated using SqueezeMeta (Tamames and Puente-Sánchez, 2019).
